# A Case of Delayed Non-infective Cystic Swelling at the Pacemaker Pocket Site

**DOI:** 10.7759/cureus.22299

**Published:** 2022-02-16

**Authors:** Praveen G Pai, Kirun Gopal, Nischal N Hegde

**Affiliations:** 1 Cardiology, Amrita Institute of Medical Sciences, Kochi, IND; 2 Cardiothoracic Surgery, Amrita Institute of Medical Sciences, Kochi, IND

**Keywords:** pacemaker complications, encapsulated seroma, pacemaker pocket swelling, pacemaker pocket infection, device related infection

## Abstract

With the expanding indications for device implantation, the number of cardiac implantable electrophysiological devices (CIED) being implanted has increased drastically. In a patient presenting with swelling at the pacemaker pocket site several years after the implantation, an infective collection due to device seeding by blood-borne microorganisms is the first diagnosis that is commonly considered. Once the diagnosis of CIED infection is made, complete removal of all the hardware is usually performed. We are describing an unusual case of a 70-year-old male with a permanent pacemaker implanted 8 years ago, who came with insidiously growing swelling at the pacemaker pocket site. He was afebrile. On examination, the swelling was soft and mobile and had no signs of inflammation. Blood cultures after 3 days of incubation did not show any growth. Ultrasound examination revealed a cystic swelling with thick septations. CT showed features suggestive of a seroma measuring 6.7 x 9.4 x 11 cm. Antibiotics were given empirically. A total of 100ml of serosanguinous fluid was drained and the pocket wall was excised. Pulse Generator (PG) was placed back into the pocket and the leads were reconnected. Culture and sensitivity testing of the drained fluid and excised tissue did not show any growth and microscopy revealed no abnormal cells. The patient was followed up on a regular basis for six months. There was no recurrence of swelling at the pacemaker site. Even though an infective abscess is the commonest cause of pacemaker pocket swelling, a non-infective swelling, however rare, must be considered as a non-infective swelling does not require complete removal of the hardware.

## Introduction

With the expanding indications for device implantation, the number of cardiac implantable electrophysiological devices (CIED) being implanted has increased drastically. In literature, pacemaker pocket infections have been classified into different types, and one such type is an abscess, presenting as a swelling at the pacemaker insertion site [1]. In a patient presenting with swelling at the pacemaker pocket site several years after the implantation, an infective collection due to device seeding by blood-borne microorganisms is the first diagnosis that is commonly considered. Once the diagnosis of CIED infection is made, complete removal of all the hardware is usually performed [1]. Non-infective swelling at the pacemaker pocket site occurs commonly within a few days after implantation and is mostly due to hematoma. Seroma occurring several years post permanent pacemaker implantation is a rare entity.

## Case presentation

**Figure 1:**
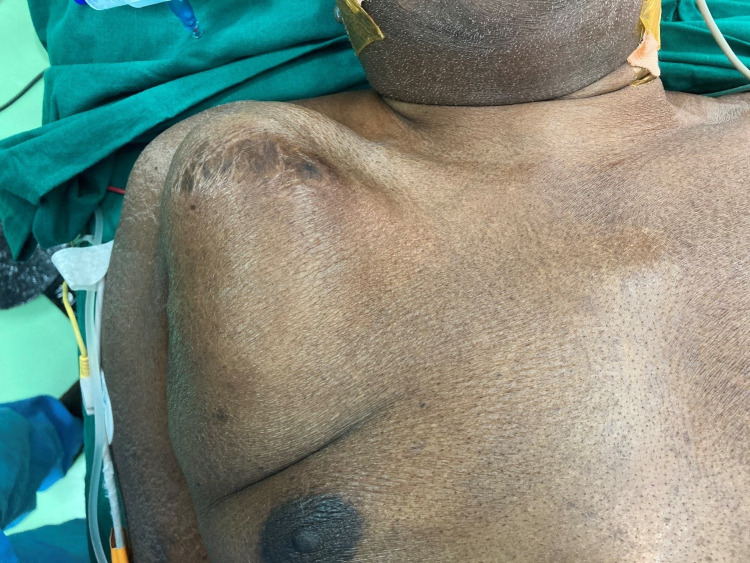
Swelling at the pacemaker pocket site

A 70-year-old male patient with multiple co-morbidities such as diabetes, systemic hypertension, dyslipidemia, hyperuricemia, allergic bronchitis, mild renal dysfunction, and moderate to severe left ventricular dysfunction with normal coronaries, had undergone dual chamber permanent pacemaker implantation on the right side for symptomatic 2:1 Atrioventricular (AV) block with right bundle branch block (RBBB) in 2008. In 2016, he underwent a pulse generator (PG) change as the pacemaker had reached its elective replacement indicator (ERI). The patient was on regular yearly follow-up. In January of 2020, a small swelling of approximately 2x3 cm was noticed at the pulse generator site without any signs of infection or inflammation. The patient was afebrile and all inflammatory markers were normal. All device and lead parameters were normal. On follow-up in July 2020, the swelling had remained static. During the review in January 2021, the swelling had increased in size to about 11 x 7 x 5 cm on gross examination (Figure 1). The patient was afebrile. The swelling was soft to feel and was not fixed to the underlying muscle plane. There were no signs of inflammation.

**Figure 2:**
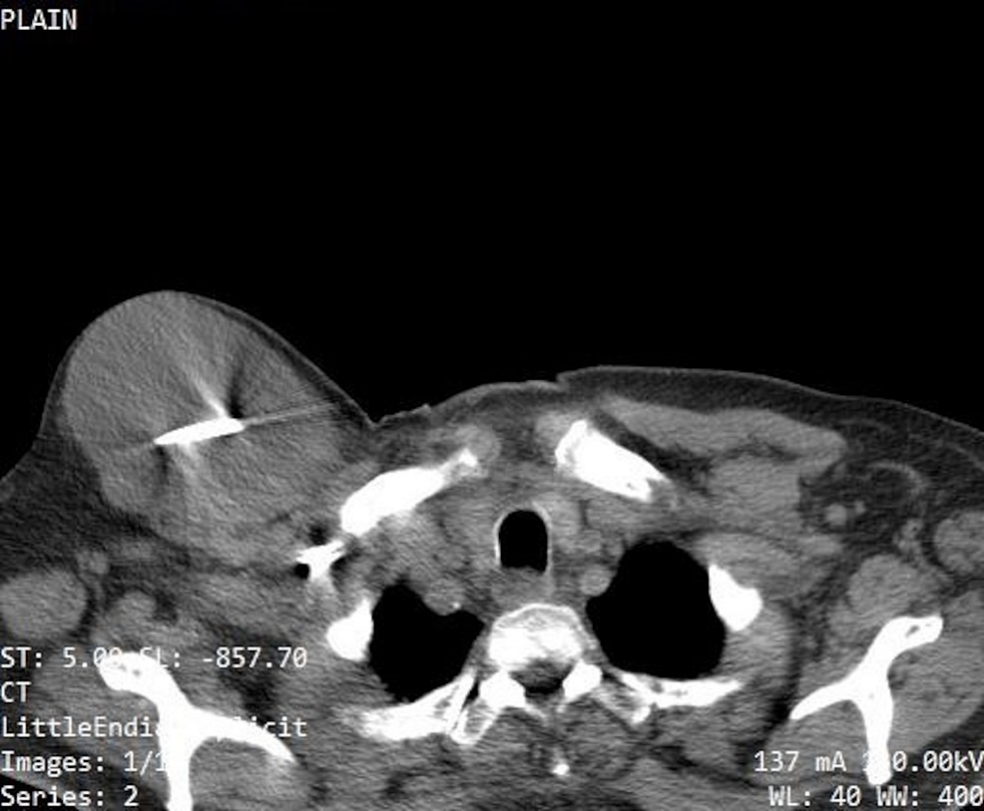
CT scan showing collection/seroma measuring 6.7 x 9.4 x 11 cm in the pacemaker insertion site. Pacemaker generator and lead wires seen within it.

His white blood cell (WBC) count was 6100 cells/µL with 68.8 % neutrophils, 18.1 % lymphocytes, and 9.6% monocytes and other inflammatory markers were normal. Both aerobic and anaerobic blood cultures did not show any growth after 72 hours of incubation. Ultrasound examination revealed the swelling to be largely cystic with multiple internal echoes and thick septations. MRI was not done as the pacemaker was not MRI compatible. CT revealed a collection/seroma measuring 6.7 x 9.4 x 11 cm (Figure 2).

Swelling at the pacemaker pocket site could be either due to an infective or a non-infective cause. The initial diagnosis considered was an infected abscess as this is the most common cause of swelling at the pacemaker pocket site. However, the patient was afebrile, WBC counts were normal with normal polymorphs, ESR and CRP were normal, and blood cultures were negative. There are only a few non-infective causes of swelling at the pacemaker pocket site which includes hematoma, seroma, and oncogenesis. Hematoma occurs within a few days post-implantation. Hence the differential diagnosis was narrowed down to either a seroma or a tumor. The swelling was cystic to feel and based on the USG and CT, seroma was the probable diagnosis. 

**Figure 3:**
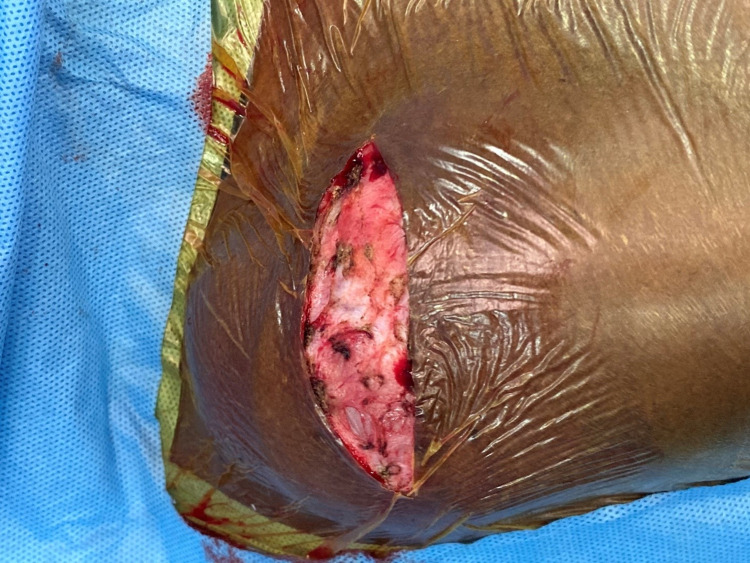
Pacemaker pocket after draining serosanguinous fluid

**Figure 4:**
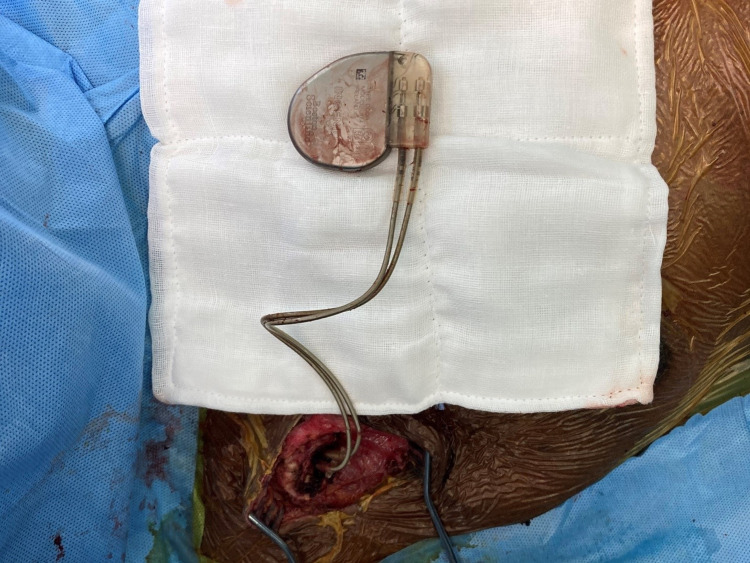
The pocket wall was excised sub-totally except for the area around lead entry into the vein. The pulse generator and the leads can be seen. The same pulse generator with the same leads were placed back into the pocket.

Even though the patient was afebrile and all inflammatory markers were normal and blood cultures were negative, antibiotics were given empirically and the option of PG change and lead explantation was given to the patient, but the patient did not consent for it. Hence, it was decided to excise the pocket wall and drain the seroma, and re-implant the PG into the same pocket. Approximately 100ml of serosanguinous fluid was drained from the pacemaker pocket and the pocket wall was excised sub-totally except for the area around lead entry into the vein. A thorough wash was given. The same PG was placed back into the pocket and the leads were reconnected (Figure 3, 4).

The drained fluid and the excised pacemaker pocket wall were sent for, both aerobic and anaerobic culture and sensitivity testing. After 7 days of incubation, no growth was seen. Microscopic examination of the excised tissue showed no abnormal cells and no organisms were seen. Device interrogation was done and all device and lead parameters were normal. The post-procedure was uneventful and the patient was discharged.

The patient was followed up on a regular basis for six months. There was no recurrence of swelling at the pacemaker site. Device interrogation showed normal device and lead parameters. The patient is currently doing well.

## Discussion

A seroma is a collection of serous fluid that develops following surgical procedures in which anatomical dead spaces have been created. Several mechanisms have been proposed such as disruption of the lymphatic drainage and blood vessels, creation of dead space, and shearing forces between the superficial hypodermis and the underlying fascia [2]. A typical lesion in which seroma occurs is the Morel-Lavallée lesion (MLL), first described in 1853 by Maurice Morel-Lavallée [3]. After surgery or a procedure that separates the hypodermis from the underlying fascia, a disruption of lymphatics and blood vessels occurs which leads to the accumulation of blood, lymph, and necrotic debris in the cavity created between the superficial and the deep tissues [2]. Over time, the blood is reabsorbed and substituted with serosanguinous fluid [2]. Finally, an inflammatory reaction leads to the formation of a pseudo capsule as the lesion becomes peripherally surrounded by fibrous tissue [2].

Swelling at the pacemaker pocket site could be either due to infective collection or a tumor or a non-infective collection, with the infective cause being the most common. Early infections after pacemaker implantation are thought to result from wound contamination at the time of surgery and most of these seem to happen within the first sixty days after the implantation [4]. Non-infective early collections are mostly due to hematoma formation. An unusual case of chylous fluid collection resulting in a swelling at the pacemaker pocket site 6 days post procedure has been reported in the literature [5]. Late infective pocket swellings are secondary infections caused by device seeding by blood-borne organisms [4]. However, late non-infective pocket swellings are rarely encountered. There are a limited number of case reports of oncogenesis occurring at the pacemaker pocket site presenting as pacemaker pocket swelling, years after the implantation [6]. Seroma occurring several years after the pacemaker implantation is very rarely reported in the literature. Small seromas can be managed conservatively by aspiration followed by compression bandaging. Large or recurring seromas require surgical management to remove the necrotic material.

## Conclusions

In conclusion, our patient presented with an insidiously progressing non-infectious swelling at the pacemaker implantation site 5 years after the PG replacement which turned out to be a seroma that is of rare occurrence. Even though infective abscess is the most common cause of pacemaker pocket swelling, other differentials, however rare, must be considered. A non-Infective cause does not require complete removal of the hardware. Whenever in doubt, a PET-CT scan can be used to distinguish between infective and non-infective cause.
